# Gender Differences in Loneliness – Four Decades of Evidence from the Framingham Heart Study

**DOI:** 10.1093/geroni/igaf122.3944

**Published:** 2025-12-31

**Authors:** Mitzi Gonzales, Matthew Scott, Alexa Beiser, Allison Mays, Joel Salinas

**Affiliations:** Cedars-Sinai Medical Center, Los Angeles, California, United States; Boston University, Boston, Massachusetts, United States; Boston University, Boston, Massachusetts, United States; New York University Grossman School of Medicine, New York, New York, United States; New York University Grossman School of Medicine, New York, New York, United States

## Abstract

Technological developments over the past four decades have altered social communication, potentially shaping experiences of loneliness. We evaluated loneliness prevalence between ages 25–94 years in men and women in the Framingham Heart Study from 1980-2019. Loneliness was assessed with the Center for Epidemiologic Studies-Depression scale. Analyses included 14,261 responses from men and 17,625 from women. Women were more likely than men to report loneliness at least one (23.7% vs. 17.5%) and ≥3 days (8.8% vs. 5.6%) per week. Using the average prevalence across four decades, 6.7% of men ages 25-34 reported loneliness ≥3 days weekly, which declined to 4.6% at ages 65–74, then rose to 7.3% at ages 75-84, and rose sharply to 12.9% at ages 85–94. Among women, prevalence remained stable from ages 25–34 (6.3%) through 54–64 (6.5%), but increased at ages 65-74 (8.7%), 75–84 (14.5%) and 85–94 (17.4%). At ages 75–84, loneliness prevalence (≥3 days/week) was twice as high in women than men (14.5% vs. 7.3%). Loneliness ≥3 times weekly in men peaked in 1980–1989 (8.6%) and then remained stable from 1990-1999 through 2010-2019 at 4.9–5.2%. In women, prevalence declined from 15.6% in 1980–1989 to a low of 5.5% in 2010–2019, a level comparable to men in this period (4.9%). In summary, women reported higher prevalence of loneliness than men and may be particularly vulnerable earlier in late life. Encouragingly, loneliness declined over the past four decades with the gap between men and women narrowing.

